# From financial lock-in to resilience reconstruction: Climate risk, internal capital markets and organizational resilience of high-carbon enterprises

**DOI:** 10.1371/journal.pone.0337896

**Published:** 2026-01-09

**Authors:** Ning Wang, Fengjuan Wang, YiTian Li, Yang Yuan

**Affiliations:** 1 Business School, Beijing Technology and Business University, Beijing, China; 2 School of Digital Finance, Beijing Institute of Economic and Management, Beijing, China; 3 School of Economic and Management, Beijing Polytechnic University, Beijing, China; 4 School of Marxism, Beijing Institute of Economic and Management, Beijing, China; Southwestern University of Finance and Economics, CHINA

## Abstract

Amid the accelerating global decarbonization, the mechanisms through which climate risk erodes the organizational resilience of high-carbon enterprises remain underexplored. Drawing on the financial lock-in perspective, we compile a panel of Chinese A-share listed enterprises in high-carbon industries from 2008 to 2023. The measurement of climate risk is achieved through the utilisation of a text-based index, with the analysis being conducted via fixed-effects models and mediation tests. This approach is employed to trace the causal pathways and to examine the moderation of these pathways by internal capital markets. The empirical results reveal three principal findings: Firstly, climate risk triggers a dual-path financial lock-in effect through financing constraints and internal capital misallocation, collectively accounting for 85.17% of the total negative impact on organizational resilience. Secondly, internal capital markets demonstrate a paradoxical dual role: while active resource reallocation buffers short-term climate shocks, financialization spillovers among subsidiaries exacerbate long-term transition risks. Thirdly, heterogeneity analysis uncovers temporal dynamics — chronic climate risks impose more severe resilience erosion than transition or acute risks, with climate-induced shocks exerting disproportionately negative effects on long-term performance relative to short-term volatility. This study offers an innovative perspective on the dynamic mechanisms of financial lock-in in the context of climate risks, proposing actionable policy solutions including the establishment of a climate-adaptive financial system, the implementation of comprehensive supervision of internal capital allocation, and the establishment of an industry-wide collaborative governance platform. These findings advance both theoretical understanding and practical strategies for managing the low-carbon transition challenges of high-carbon enterprises.

## 1. Introduction

**Fig 1:**
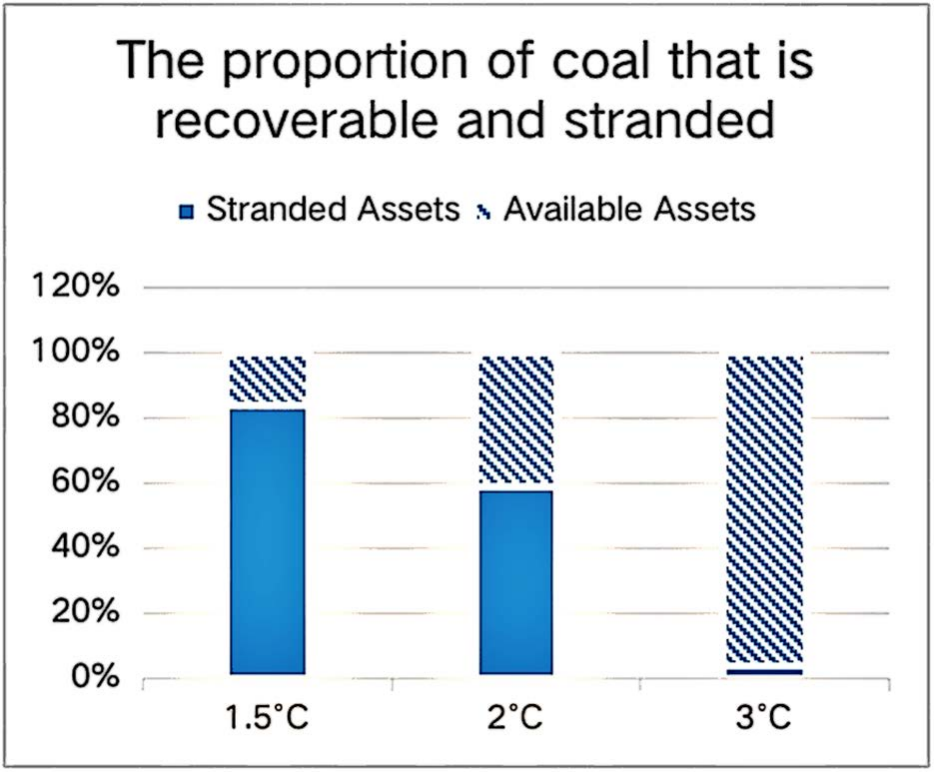
Proportion of stranded assets among coal mining assets under different warming targets.

As global climate risks intensify, corporations confront dual pressures from physical and transition risk exposures. Physical risks manifest through asset impairment and operational disruptions caused by escalating extreme weather events. Empirical evidence from the World Meteorological Organization (2021) [1], indicates that climate-driven extremes have contributed to 50% of global disaster-related fatalities and economic losses over the past half-century. Insurance industry analyses further quantify this vulnerability, estimating $270 billion in global climate-related economic damages for 2022 alone. It is estimated that high-carbon enterprises will absorb 62% of these losses. [2,3]. Transition risks manifest as stranded asset crises triggered by abrupt policy changes. According to the Financial Times’s forecast data, shown in Fig 1,if the temperature rise target is kept below 2°C, approximately 59% of global coal reserves will become stranded assets. If the target is set at 1.5°C, however, only 16% of coal reserves will remain recoverable. Paradoxically, the response of financial institutions to climate transition remains slow. Nearly 60% of banks allocate less than 5% of their loan portfolios to climate-related investments, and over a quarter of institutions have yet to establish climate financing mechanisms World Bank (2023) [4]. Such transition-capital decoupling entrenches high-carbon enterprises in a dual predicament: incurring escalating compliance costs from climate regulations while being systematically excluded from green financing channels.

**Fig 2:**
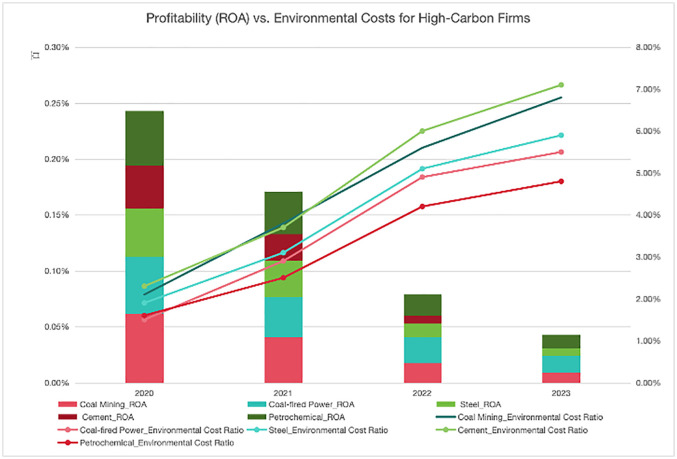
Comparison between profitability (ROA) and environmental costs of high carbon enterprises. Data sources: National Bureau of Statistics, Chinese Academy of Environmental Sciences, Wind database (as at December 2023).

As the world’s largest carbon-emitting economy (accounting for approximately one-third of global emissions), China’s “dual carbon” strategy exhibits three distinct characteristics: institutional rigidity, compressed timelines, and capital restructuring. At the institutional level, state-owned capital exerts significant control over high-carbon industries. According to data collected by the author and made available to the public, within the top five industries in terms of carbon emissions, sample data shows that 83% of high-carbon enterprises are state-owned entities. This phenomenon can be attributed to the attainment of an asset specificity index that has reached a critical threshold of 0.72, in conjunction with a 24% burden of redundant personnel employment. With regard to temporal trends, data from the National Development and Reform Commission reveals a 3.8% year-on-year decrease in national energy intensity and a 3.4% year-on-year decrease in carbon emissions intensity in 2024. These figures are equivalent to three times the average of OECD countries. This precipitous policy transition resulted in a marked increase in corporate compliance costs. As demonstrated in Fig 2, between 2020 and 2023, the average return on assets for high-carbon industries decreased from 4.9% to 0.9%, while environmental management costs as a percentage of operating revenue increased from 1.9% to 6.1% over the same period. This indicates that high-carbon enterprises experienced a decline in performance volatility and resilience from both a cost and a benefit perspective. With regard to capital allocation, the presence of carbon risk premiums and market inefficiencies in transition finance has been demonstrated to result in an approximate 50% increase in the default probability of high-emission enterprises (Xu Ruizhe et al.,2024) [5]. Furthermore, commercial banks have demonstrated a declining propensity to extend credit to high-emission enterprises (Liu Lianbiao et al., 2025) [6]. In their 2020 study, Ma Jun et al. [7] estimated that, under the 2°C scenario, the probability of default among China’s major coal-fired power companies will rise to 22% by 2030, due to declining demand and rising financing costs. This high-intensity, compressed transition path—which is far more intense than the gradual transition paths of other countries—provides an extreme scenario for observing the financial lock-in effect.

The financial lock-in theory is a significant departure from the technodeterminist limitations of the traditional carbon lock-in paradigm, providing a financial perspective for studying the impact of climate risks on corporate organizational resilience. While Unruh’s (2000) [8] seminal work on carbon lock-in offers a framework for understanding the path dependence in the formation of the technology-institutional complex, it does not address the reinforcing mechanisms of financial capital on high-carbon pathways. The financial lock-in theory integrates Williamson’s (1975) [9] asset specificity theory, Diamond’s (2000) [10] financial intermediation theory, and Gennaioli et al.’s (2015) [11] behavioral cognitive framework to construct a “financing-behavior” rigid mechanism. The specificity of assets has been demonstrated to engender sunk cost barriers, increase transaction costs, and impede the exit path for high-carbon assets. Furthermore, the presence of climate risk premiums has been shown to result in elevated costs associated with debt financing, thereby impeding the reinvestment capabilities of affected firms. The risk perception biases of management serve to reinforce the high-carbon path dependence that is characteristic of the present context, thereby forming a feedback loop that is resistant to disruption. The interplay of these dual mechanisms collectively constitutes the core pathway for the erosion of micro-organizational resilience. A close examination of the lock-in effect and organizational resilience theory reveals a profound interconnection between these two concepts. Conventional resilience research underscores the cyclical relationship between emergency response capabilities, adaptability, and risk [12]. However, this research neglects to address the profound constraints imposed by capital allocation rigidity on organizational resilience. The utilization of internal capital markets by firms to mitigate climate risk shocks has been demonstrated to result in short-term resource patches (Baker & Nelson, 2005) [13]. However, herd effects have been shown to amplify system vulnerability through signal contagion (Bikhchandani et al., 1992) [14], leading to resource reallocation that deviates from the innovation track. The present study seeks to contribute to the ongoing discourse by investigating the role of the internal capital market as either a risk buffer or a driver of financialization. The operation of the lock-in effect and organizational resilience theory remains unclear in extant research.

The present study focuses on a critical challenge: The present study explores the manner in which climate risk is transmitted through financial lock-in mechanisms, thereby undermining organizational resilience within high-carbon enterprises. Our analysis has yielded three key contributions to the extant literature: Firstly, we innovatively apply text mining techniques to construct a climate risk lexicon from corporate disclosures and policy documents. This enables systematic assessment of resilience impacts previously obscured in unstructured data. This approach enhances the measurement precision beyond conventional financial metrics. Secondly, we unveil a theoretical paradox in the functioning of internal capital market (ICM) functions: While active ICMs offer short-term protection against climate-related shocks by facilitating resource reallocation, their propensity for financialization, particularly the behaviors associated with subsidiary herding, can exacerbate capital misallocation, leading to long-term adaptation challenges. This finding provides a resolution to the conflicting evidence regarding ICM efficiency under transition risks. Thirdly, the dynamic pathways through which financial lock-in operates are extended as follows: (1) external financing constraints amplify climate vulnerability through credit rationing, and (2) internal capital misallocation reinforces strategic rigidity through sunk-cost escalation. This dual mechanism framework extends lock-in theory to organizational resilience research, offering actionable insights for high-carbon transition governance.

## 2. Literature review and research hypothesis

### 2.1 Theoretical evolution: From carbon lock-in to financial lock-in

The traditional carbon lock-in theory, underscores the notion of path dependence in the formation of the so-called “technology-institutional complex.” This theoretical framework highlights that high-carbon industries become entrenched in unsustainable development patterns, a phenomenon attributed to the rigidity of technology and the inertia of institutions. The concept of path dependence finds manifestation in various sectors, including power [15], transportation [16], and construction [17]. The perpetuation of existing technological systems is facilitated by rent-seeking and regulatory capture, leading to the formation of “social inertia” that hinders the dissemination of alternative technologies. While this framework reveals structural barriers at the macro level, it overlooks the micro-level drivers of capital allocation. As research has progressed, scholars have initiated the development of indicator systems to quantitatively assess the degree of carbon lock-in, with a primary focus on factors such as industry, institutions, and technology. Unruh et al. [8] investigated the phenomenon of carbon lock-in, exploring its implications through a multifaceted analysis encompassing fixed asset investment, technological lock-in, institutional lock-in, and social behavioral lock-in. However, their computational methods are primarily used to calculate carbon lock-in at the macro level, such as the national or regional level, and struggle to delve into the micro level of individual enterprises. Moreover, the theoretical framework of technological lock-in does not adequately address the rationale behind enterprises’ sustained investment in traditional assets, despite the awareness that high-carbon pathways are inherently unsustainable.

The concept of financial lock-in integrates the specificity of assets, financing constraints, and behavioral decisions to construct a rigid triangular framework of “assets-financing-behavior.” The theory of asset specificity proposed by Williamson [18] is given new meaning in this study. High-carbon assets possess a high degree of specificity, with their physical form and functional design firmly embedded within high-carbon industries. Assets characterized by specificity are often characterized by suboptimal liquidity, and are frequently utilized by internal institutions, thereby entering a cycle of defensive investment [19]. According to Douglas Diamond’s financial intermediation theory model, the presence of information asymmetry has the effect of distorting corporate financing capabilities. This theoretical framework provides a foundation for the analysis of the impact of transition risks on financing costs. Significant risk premiums are imposed on high-carbon industries by creditors to hedge against policy risks, such as rising carbon prices. Research [20] indicates that the difficulty and cost of financing projects like coal-fired power plants compel companies to divert low-carbon transition budgets to maintain traditional capacity. This results in a “defensive lock-in” oriented toward short-term survival. Cognitive biases provide a complementary explanation of financial lock-in from a behavioral decision-making perspective at the micro level. Gennaioli et al. [11] propose a behavioral framework indicating that ambiguity in policy signals triggers risk-averse psychology among management, leading them to prioritize investments in traditional businesses with predictable returns, thereby forming a “high-carbon comfort zone” at the cognitive level.

Existing carbon lock-in theory analyses consider fixed asset investment and institutional factors but lack explanations within the framework of corporate finance and behavioral economics. The theory of asset specificity has evolved from a static “sunk cost barrier” to a dynamic “risk premium generator.” The theory of financing constraints has shifted from generalized credit rationing to “defensive capital misallocation” under climate policy coordination. Furthermore, behavioral decision-making mechanisms have transitioned from rational expectations to “endogenous lock-in reinforcement” triggered by policy cognitive biases. This paradigm shift unveils the micro-level drivers that underpin firms’ active participation in deepening lock-in.

### 2.2 Mechanism deepening: Climate risk erodes organizational resilience in high-carbon enterprises through financial lock-in

#### 2.2.1 Climate risks erode organizational resilience.

Climate risks encompass both physical climate risks and climate transition risks. These risks have the potential to compel entities to enter a state of financial lock-in, characterized by rigid asset structures and financing constraints that impede the transition of businesses toward low-carbon practices. The physical risks manifest as extreme weather events, which cause impairment to high-carbon assets [21,22]. This leads to a decline in collateral value [23], resulting in rising insurance costs. Consequently, commercial banks tighten credit conditions [24,25], forcing companies to rely on internal funds to maintain traditional production capacity. This process forms defensive lock-in [26]. Transition risks emerge from the ambiguity surrounding carbon policies, the evolution of carbon market regulations, and the recalibration of emission quotas, which diminish companies’ propensity to invest in low-carbon technologies [27]. Management teams have a consistent history of injecting internal capital into traditional businesses as a means of hedging against policy shocks, a practice that has been shown to trigger strategic lock-in [28]. Collectively, these mechanisms contribute to the erosion of organizational resilience—defined as a company’s capacity to maintain stability and adapt to external shocks.

Organizational resilience is defined as an organization’s capacity to maintain stability and swiftly revert to its original state when confronted with external shocks and complex, ambiguous situations. This ability is considered the core capability for crisis response [29]. Organizational resilience is the core capability for high-carbon enterprises to address climate risks, primarily manifested in three dimensions of resilience: stability, adaptability, and evolvability [30]. Organizational resilience faces unique challenges in high-carbon enterprises. Enterprises with a high carbon footprint are acutely affected by policy changes. The variability in environmental costs resulting from the dual carbon goals and the pressure to restructure supply chains [31,32] complicates their transformation process. At present, the financial demands of typical carbon capture initiatives are considerable, and avenues for funding are severely restricted, with a heavy reliance on corporate self-financing and government subsidies, and an absence of participation from social capital. Enterprises with a high carbon footprint encounter substantial obstacles when attempting to obtain policy support for their transformation initiatives. Therefore, a fundamental contradiction emerges between financial lock-in and organizational resilience. Companies sacrifice long-term evolutionary capabilities in pursuit of short-term stability. This ultimately leads to a rigid, closed loop of high-carbon path dependence amid escalating climate risks.

In summary, climate risks have been shown to have a significant impact on the organizational resilience of high-carbon enterprises, resulting from the combined effects of physical risks and transition risks. Therefore, hypothesis H1 is proposed.

**H1 Climate risks reduce the organizational resilience of high-carbon enterprises.**

#### 2.2.2 The intermediary role of financial lock-in.

Climate risks have been shown to exacerbate financial lock-in through external financing constraints and internal capital misallocation, thereby weakening the organizational resilience of high-carbon enterprises. Financing constraints are associated with the acquisition of external capital, while resource misallocation is associated with the internal allocation of capital. On the one hand, the exclusion of external green financing, coupled with credit policies that lack tolerance for short-term innovation failures, can lead to a “crowding-out effect” (Guo & Fang, 2024) [33], such as banks restricting credit to the coal industry and the depreciation of collateral value, forcing companies to rely on internal funds to maintain high-carbon operations. Conversely, in circumstances characterized by short-term cash flow constraints, managerial decisions prioritize capital, labor, and other resources away from enterprises with the highest marginal output. This prioritization, in turn, results in resource misallocation. Empirical research has confirmed that the marginal output difference between China and India is 50%−100% higher than that of the United States (Hsieh & Klenow, 2009) [34]. The allocation of funds to high-carbon sectors has been shown to distort resource allocation by crowding out innovation (Hong Lianyong et al., 2025) [35]. Furthermore, an increase of one unit in the coal industry’s financing constraint index corresponds to a 64-unit decrease in green innovation levels [36], which in turn has the effect of crowding out investments in low-carbon technologies. Concurrently, constraints in financing have been shown to intensify the discord between shareholders and creditors. The application of agency theory in the context of high-risk investments, such as low-carbon projects, has been identified as a key factor in the restriction of such investments through contractual terms imposed by creditors [37]. Within the context of corporate groups, agency issues have the potential to give rise to rent-seeking behavior [38]. This phenomenon can compel the adoption of inefficient mature technologies rather than cutting-edge technologies, thereby leading to resource misallocation. This cycle of high-carbon investment→financing constraints→reinvestment in high-carbon sectors forms an intermediary path leading to resilience loss. In summary, this paper proposes Hypothesis H2:

**H2 Climate risk reduces organizational resilience of enterprises through financial lock-in mechanisms, with financing constraints and internal capital misallocation serving as primary pathways.**

### 2.3 The dual-edged role of internal capital markets

#### 2.3.1 The impact of the peer effect of internal capital market scale.

As different business units under the control of the same corporate group, the interconnections among group member companies primarily manifest in business transactions and capital flows. The former includes mergers and acquisitions, asset sales, and commodity trading (Cheung et al., 2006 [39]), while the latter is realized through equity transfers, dividend payments, loans, and mutual guarantees (Powell et al., 2008 [40]). Extensive research has confirmed that internal capital markets play a significant role in strengthening financial ties between subsidiaries of corporate groups in emerging market economies and mitigating the impact of constrained external financing conditions (Shackman, 2007 [41]; Li and Rwegasira, 2010 [42]; Yan et al., 2010 [43]; Tan et al., 2018 [44]).Compared to external investors, group headquarters possess a deeper understanding of subsidiary operations, which can mitigate investment distortions caused by information asymmetry. ICM enhances overall efficiency by consolidating dispersed cash flows and channeling funds toward high-return projects, such as strategic transformation investments.

An active internal capital market mitigates climate risk through two mechanisms: substituting external financing and optimizing intertemporal resource allocation. First, cross-sectoral capital pools enable sustained investment in low-carbon sectors, offsetting constraints from green credit restrictions [45]. Second, dynamic adjustment of asset allocation proportions [46] helps diversify policy-induced regulatory risks. These mechanisms enhance corporate resource allocation flexibility, thereby attenuating climate risk’s detrimental effects on organizational resilience. Therefore, Hypothesis H3a is proposed:

**H3a The increase in internal capital market activity can reduce the negative impact of climate risk on the organizational resilience of coal enterprises.**

#### 2.3.2 The impact of the peer effect of internal capital financialization.

The internal capital market within a firm adjusts exits based on productivity differences and reallocates market shares. Critics have highlighted efficiency losses attributable to agency problems and rent-seeking behavior. Rajan (2010) [47] discovered that corporate groups tend to transfer resources from high-return sectors to inefficient sectors to maintain power balance, leading to “egalitarian” allocation. In the Chinese context, some firms have allocated internal funds to non-core financial assets, such as real estate and trusts, thereby initiating a “decoupling from the real economy” [48]. This phenomenon of financialization exerts a dual influence on organizational resilience, manifesting through two distinct channels. Firstly, it fosters signal contagion, wherein information-disadvantaged follower firms tend to emulate the financialization behaviors exhibited by information-advantaged leader firms, thereby intensifying the industry’s proclivity for short-term profit-seeking [49]. Secondly, it engenders resource crowding, leading to the consumption of cash flow through non-core investments [50], consequently impeding the capacity to transition to low-carbon operations.

The financial behaviors of subsidiaries within corporate groups generate peer effects, thereby creating contagion effects [51] Industry-wide financialization exacerbates resilience erosion through resource crowding-out and signal distortion mechanisms. On the one hand, non-core business investments deplete cash flows, constrain low-carbon technology R&D, and displace productive capital through enterprise financialization [52] On the other hand, herding effects propagate industry decline expectations, incentivizing excessive financial asset investments that elevate external financing costs [53]. Simultaneously, internal capital overinvestment distorts market competition mechanisms, degrading internal capital market efficiency. This dual mechanism not only undermines companies’ innovation and transformation capacities but also amplifies climate risk’s detrimental impacts on organizational resilience. Therefore, Hypothesis H3b is proposed:

**H3b The financialization of the internal capital market and the peer effect can increase the negative impact of climate risk on the organizational resilience of coal enterprises.**

**Fig 3:**
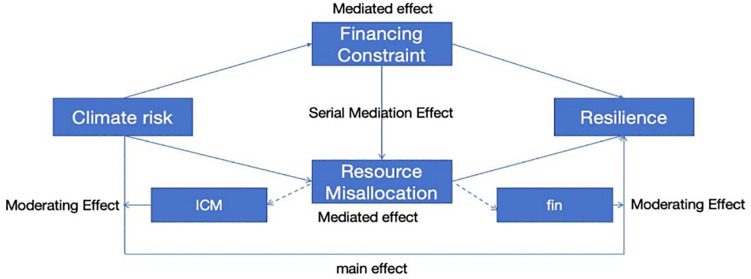
Graphical representation of the hypothesis for the study.

The interrelationships among the various assumptions are illustrated in Fig 3. Climate risk has been demonstrated to weaken the resilience of business organizations through financial lock-in, with the financial lock-in pathway consisting of external financing constraints and internal capital misallocation, which may involve chain intermediation. The scale of the internal capital market and financialization peer effects act as moderating pathways to regulate internal allocation efficiency.

## 3. Study design

### 3.1 Samples and data

To ensure financial data consistency under China’s 2007 New Corporate Accounting Standards, our analysis focuses on A-share listed companies from the Shanghai and Shenzhen stock exchanges during 2008–2023. We exclude independent enterprises, retaining only group-affiliated entities to investigate how corporate financialization influences mature internal capital markets. Enterprise group membership is determined using Cai Weixing et al. (2023) [54]’s criteria: A listed company qualifies as group-affiliated if its largest shareholder is either (1) a formally registered group company or (2) an entity functionally equivalent to a group parent company. Following Zhang Mingming et al. (2024) [55] we employ carbon emission models and industry-specific carbon intensity benchmarks to identify high-carbon sectors. Industries exceeding the carbon intensity index threshold (including coal, petrochemicals, and related sectors) are classified as high-carbon industries. The final sample consists of 2,053 firm-year observations across 233 high-carbon enterprise groups. All financial and governance data are obtained from the CSMAR database.

### 3.2 Variable definition

#### 3.2.1 Independent variable: Climate risk.

This study measures corporate climate risk exposure through text analysis of annual reports. Data acquisition commenced with downloading Management Discussion and Analysis (MD&A) sections from A-share listed companies’ annual reports (2008–2023) via the Juchao Information Network. Lexicon construction integrated three complementary sources: (1) climate risk-related expressions extracted from MD&A texts, (2) meteorological disaster taxonomies published by the National Meteorological Science Data Center, and (3) historical case references from the China Meteorological Disaster Yearbook. To enhance lexical objectivity, machine learning augmentation was implemented following Du et al.(2023) [56], the methodology employed machine learning techniques to train the annual report corpus. Utilizing the word vector CBOW model, the ten most semantically proximate terms to the seed word set were identified, thereby expanding the initial lexicon. This iterative refinement process established the definitive “climate risk” terminology repository. The climatic risk metric was subsequently quantified as the ratio of aggregated frequency counts for the expanded climate risk lexicon relative to the total lexical volume within management discussion and analysis (MD&A) sections. Firms exhibiting higher metric values demonstrate statistically greater susceptibility to climate-related risks.

#### 3.2.2 Dependent variable: Organizational resilience.

Following Wu Xiaobo (2022) [57], organizational resilience is conceptualized as a bidimensional construct comprising growth and volatility. Growth is operationalized as the three-year cumulative sales revenue growth denoted as Growth; volatility is quantified by the annualized standard deviation of monthly stock returns, denoted as Volatility. The composite organizational resilience index is ultimately synthesized via the entropy weighting method. The entropy method resolves the negative correlation between growth and volatility indicators, thereby ensuring greater consistency between the composite resilience score and the duality theory of “stability-agility”.

#### 3.2.3 Mediating variables.

(1) Financing Constraints (*SA*)

Financing constraints (SA index) according to Hadlock (2010) [58] financing constraints are quantified using the Size-Age (SA) index, constructed from exogenous firm characteristics to mitigate endogeneity: SA_*i,t*_*=−0.737 × Size*_*i,t*_* + 0.043 × Size*^*2*^_*i,t*_* − 0.040 × Age*_*i,t*_

where Size_i,t_ denotes the natural logarithm of total assets, and Age_i,t_ represents the years since firm establishment.

(2) Internal Capital Market Efficiency(*AbRpt*)

Adapting Richardson (2006) [59], ICM efficiency is proxied by the absolute deviation between actual and predicted internal capital transactions. The prediction model is specified as: *ICM*_*i,t*_* = α*_*0*_* + α*_*1*_*ICM*_*i,t-1*_* + α*_*2*_*TobinQ*_*i,t-1*_* + α*_*3*_*Cash*_*i,t-1*_* + α*_*4*_*Lev*_*i,t-1*_* + α*_*5*_*Size*_*i,t-1*_* + α*_*6*_*Roa*_*i,t-1*_* + α*_*7*_*IPO*_*i,t-1*_* + λ*_*i*_* + μ*_*t*_* + ε*_*i,t*_,*ICM*_*i,t*_ denotes Internal related transaction amount in year t; TobinQ_i,t−1_ denotes Market-to-book ratio at year t − 1;IPO_i,t−1_ denotes Years since initial public offering; λ_i_ indicates Firm fixed effects; μ_t_ indicates Year fixed effects; The residual ε_i,t_ captures ICM inefficiency: positive values indicate overinvestment, negative values denote underinvestment, with larger absolute values reflecting lower efficiency. The study commences with an examination of the financing and investment functions of internal capital markets. It employs methodologies for assessing investment efficiency and constructs efficiency indicators for the internal capital markets of group member companies.

#### 3.2.4 Regulated variable.

(1) Internal Capital Market Activity(*Rptr*)

Following Tong Pan et al. (2022) [60] the agency variables within the internal capital market are calculated based on transaction data. Specifically, the activity of the internal capital market is measured by the ratio of net inflow of related transaction funds to total assets at the end of the year. This metric reflects the extent of internal capital allocation and its efficiency within the firm.

(2) Peer Effect Financialization *(Ind_Fin)*

Financialization of peer effect industry (Ind_Fin), s constructed based on the ratio of corporate financial assets to total assets, following the framework established by Li Qiumei (2020) [61]. The variable, Ind_Fin, captures the average financialization level among peer firms that share the same industry, region, and group. Specifically, peers of the focal firm i are defined as other firms within the same industry classification, registered in the same province, and operating within the same industry and regional context. The industry classification is determined according to the first code of the “Industry Classification Guidelines for Listed Companies” issued by the China Securities Regulatory Commission in 2012.

All variables are presented in Table 1.

**Table 1:** Variable list.

Variable-definition	Variable symbol	Variable declaration
explanatory variable	*Climaterisk*	The frequency of meteorological risk words was calculated by text analysis and statistical management discussion
explained variable	*Resilience*	Organizational resilience is calculated comprehensively according to the two dimensions of growth and volatility, using entropy value method
metavariable	*SA*	Financing constraints: SA index
	*AbRpt*	Efficiency of the internal capital market: abnormal trading
regulated variable	*Rptr*	Activity in the internal capital market
	*Ind_Fin*	Financialization and group effect of industry financialization and group effect of industry financialization
controlled variable	*age*	Enterprise listing age, ln (the year of the current year-the year of the listing +1)
	*roe*	Return on equity
	*lev*	Interest-bearing debt/total assets
	*size*	EBITDA/total assets
	*top1*	The shareholding ratio of the largest shareholder
	*MB*	The ratio of market value to total assets on the books
	*growth*	Total assets growth rate
	*Year*	Annual dummy variable
	*Ins*	Industry dummy variables

### 3.3 Empirical Models

The core question of this paper is how climate risk (Climate risk) affects the organizational resilience (Resilience) of high-carbon enterprises through financial lock-in mechanisms (SA and AbRpt), while considering the moderating effects of internal capital market activity (Rptr) and financial peer effect (Ind_Fin).

#### 3.3.1 Main effect.

First, construct the main effect model (1) to test the impact of climate risk on organizational resilience:


$${Resilience}_{i,t}=\alpha +\beta_{\text{1}}{Climate\;risk}_{i,t\;(t-\text{1})}+\beta_{\text{2}}{Controls}_{i,t}+\varepsilon_{i,t}$$ (1)

#### 3.3.2 Mediation effects: Financial lock-in mechanisms.

he mediation effect model (2-1) (2-2) is constructed to test the mediation role of financial lock-in mechanism:

Intermediate path 1: Financing constraints (SA)


$${Resilience}_{i,t}=\alpha +\beta_{\text{1}}{Climate\;risk}_{i,t}+\beta_{\text{2}}{SA}_{i,t}+\beta_{\text{3}}{Controls}_{i,t}+\varepsilon_{i,t}$$ (2-1)

Intermediate path 2: capital mismatch (AbRpt)


$${Resilience}_{i,t}=\alpha +\beta_{\text{1}}{Climate\;risk}_{i,t}+\beta_{\text{2}}{AbRpt}_{i,t}+\beta_{\text{3}}{Controls}_{i,t}+\varepsilon_{i,t}$$ (2-2)

#### 3.3.3 Moderating effects.

To test the moderating effect, we construct the interaction term between the moderating variable and the independent variable to construct models (3-1) and (3-2)

Regulation path 1: Internal capital market business resource allocation


$${Resilience}_{i,t}=\alpha +\beta_{\text{1}}{Climaterisk}_{i,t}+\beta_{\text{2}}{Rptr}_{i,t}+{{\beta_{\text{3}}(Climaterisk}_{i,t}{\times Rptr}_{i,t})+\beta }_{\text{4}}{Controls}_{i,t}+\varepsilon_{i,t}$$ (3-1)

Regulation path 2: Financial resource allocation in internal capital market


$${Resilience}_{i,t}=\alpha +\beta_{\text{1}}{Climaterisk}_{i,t}+\beta_{\text{2}}{Ind\_Fin}_{i,t}+{{\beta_{\text{3}}(Climaterisk}_{i,t}{\times Ind\_Fin}_{i,t})+\beta }_{\text{4}}{Controls}_{i,t}+\varepsilon_{i,t}$$ (3-2)

### 3.4 Descriptive statistics

The study sample comprises 2,053 observations, with descriptive statistics for all variables presented in Table 2. The dependent variable organizational resilience (Resilience) exhibits a mean of 0.388 (SD = 0.297), indicating moderately low overall risk resistance among sample firms within the theoretical range [0, 1]. The operational range spans [0.018, 0.887], demonstrating substantial heterogeneity in firms’ capacity to withstand external shocks. The mean of the independent variable climate risk (Climate Risk) displays a mean of 0.222 (SD = 0.151), significantly exceeding the full-sample A-share average of 0.009 calculated using identical criteria, corroborating high-carbon industries’ heightened exposure to climate transition risks.

**Table 2:** Descriptive statistics.

Variables	Obs	Mean	Std. Dev.	Min	Max
Resilience	2053	0.388	0.297	0.018	0.887
Climate Risk	2053	0.222	0.151	0.023	0.711
AbRpt	2053	−2.878	5.376	27.254	−7.011
SA	2053	−3.822	0.32	−4.561	−2.344
rptr	2053	2.069	3.04	0	17.278
Ind Fin	2053	0.085	0.084	0	0.513
age	2053	2.665	0.534	1.386	3.401
roe	2053	0.049	0.151	−0.988	0.285
size	2053	23.249	1.506	20.229	27.132
lev	2053	0.54	0.179	0.093	0.924
top1	2053	0.415	0.165	0.093	0.771
MB	2053	0.801	0.241	0.198	1.233
growth	2053	0.094	0.177	−0.213	0.959

The mediating variable capital misallocation (AbRpt) spans values from −7.011 to 27.254, with a standard deviation 1.87 times greater than its mean, revealing pronounced polarization in financial reporting practices. The financing constraint measure (SA) averages −3.822 (minimum = −4.561), consistent with prior benchmarks (Hadlock & Pierce, 2010), confirming moderate financing constraints across the sample. The financial peer effect (Ind_Fin) has a mean of 0.085, reflecting 8.5% average contagion intensity from industry-level financial asset allocations, with a right-skewed distribution (maximum = 0.513).

Control variable statistics reveal that firm size (Size) log-asset mean of 23.249 corresponds to an asset scale between RMB 460 billion and 550 billion, aligning with industry-typical financial leverage (Lev) of 54%. The market-to-book ratio (MB) of 0.801 signals market valuations below book values, consistent with high-carbon sector valuation discounts. Continuous variables generally show standard deviations smaller than means (e.g., ROE: mean 0.049 vs. SD 0.151), while ratio variables (Lev, Top1, Ind_Fin) strictly adhere to the [0, 1] theoretical range, confirming no extreme outliers.

## 4. Empirical results and analysis

### 4.1 Benchmark analysis

The benchmark analysis examines how climate risk impacts organizational resilience in high-carbon groups. Table 3 reports fixed-effects regression results. Column (1) presents the baseline specification from Model (1). Under the control of variables such as firm size (Size), debt-to-asset ratio (Lev), and cash flow (Cashflow), the regression coefficient for climate risk (Climate risk) is-0.021, with a standard error of 0.007, and is significant at the 1% level (t = −3.00). This implies a 2.1 percentage point decline in organizational resilience per standard deviation increase in climate risk. Among controls, firm size shows a positive coefficient (0.163), confirming resource endowment effects, whereas leverage ratio (−0.089) demonstrates financial rigidity’s vulnerability-enhancing role.

**Table 3:** Baseline regression results.

Variable	(1)	(2)
	Resilience	Resilience
Climate risk	−0.029***	
	(0.011)	
L. Climate risk		−0.021*
		(0.011)
age	−0.006**	−0.007**
	(0.003)	(0.003)
roe	0.004	0.002
	(0.009)	(0.009)
size	0.012***	0.011***
	(0.001)	(0.001)
lev	−0.033***	−0.036***
	(0.008)	(0.009)
top1	0.015*	0.010
	(0.009)	(0.009)
MB	−0.002	0.003
	(0.008)	(0.008)
growth	−0.004	−0.001
	(0.007)	(0.008)
Constant	0.142***	0.192***
	(0.026)	(0.027)
Observations	2,053	1,758
R-squared	0.969	0.972
Year FE	Yes	Yes
Ins FE	Yes	Yes

Standard errors in parentheses.

*** p < 0.01, ** p < 0.05, * p < 0.1.

Column (2) introduces dynamic effects through one-period lagged climate risk (L. Climate Risk). The lagged term coefficient (−0.017, p < 0.10) confirms persistent risk impacts, though 23.5% smaller in magnitude than the contemporaneous effect (−0.021). This temporal pattern justifies prioritizing current-period climate risk in subsequent analyses.

### 4.2 Mechanism analysis: Based on financial lock-in

#### 4.2.1 Financing constraint locking mechanism.

Following the three-step regression analysis based on the mediation effect model, the regression results are reported in Table 4. First, Table 4, column (1), shows that the coefficient of climate risk on organizational resilience is −0.029 (significant at the 1% level), indicating a statistically significant negative total effect of climate risk on organizational resilience. Second, the mediation path test reveals that the coefficient of climate risk on the mediator variable financing constraints (SA) is 0.055 (significant at the 1% level), confirming a positive relationship where climate risk exacerbates corporate financing constraints. Finally, the joint effect test (Model 2−1) demonstrates that after introducing both climate risk and financing constraints (SA), the coefficient of SA on organizational resilience is −0.246 (significantly negative at conventional levels), proving that financing constraints weaken organizational resilience. Notably, the coefficient of climate risk declines from −0.029 in column (1) to −0.015 (statistically insignificant), providing evidence that financing constraints fully mediate the impact of climate risk on organizational resilience. This result aligns with Hypothesis H2’s proposed financial lock-in mechanism.

**Table 4:** Financing constraint locking mechanism.

Variable	(1)	(2)	(3)
	Resilience	SA	Resilience
SA			−0.246***
			(0.023)
Climate risk	−0.029***	0.055***	−0.015
	(0.011)	(0.010)	(0.011)
age	−0.006**	0.081***	0.014***
	(0.003)	(0.002)	(0.003)
roe	0.004	0.015*	0.008
	(0.009)	(0.008)	(0.008)
size	0.012***	−0.042***	0.002
	(0.001)	(0.001)	(0.002)
lev	−0.033***	0.054***	−0.020**
	(0.008)	(0.008)	(0.008)
top1	0.015*	−0.028***	0.009
	(0.009)	(0.008)	(0.008)
MB	−0.002	0.057***	0.012*
	(0.008)	(0.007)	(0.007)
growth	−0.004	0.027***	0.003
	(0.007)	(0.007)	(0.007)
Constant	0.142***	2.025***	0.639***
	(0.026)	(0.024)	(0.053)
Observations	2,053	2,053	2,053
R-squared	0.969	0.703	0.971
Year FE	Yes	Yes	Yes
Ins FE	Yes	Yes	Yes

Standard errors in parentheses.

*** p < 0.01, ** p < 0.05, * p < 0.1.

The indirect effect of climate risk on organizational resilience through financing constraints is calculated as 0.055×(−0.246) =−0.0135, accounting for (−0.0135)/ (−0.029)≈ 46.55% of the total effect. Economically, a one-unit increase in climate risk reduces organizational resilience by approximately 0.0135 units through the financing constraint channel, representing 46.55% of the total negative impact. These findings suggest that high-carbon enterprises are trapped in a self-reinforcing cycle of “high-carbon investment→financing constraints→reinvestment in high-carbon activities,”thereby perpetuating path-dependent carbon lock-in.

#### 4.2.2 Resource misallocation.

The regression results in Table 5 demonstrate that climate risk undermines enterprise organizational resilience by impairing internal capital market efficiency (Abrupt), with the mechanism analyzed through the following evidence: Column (1) reveals a statistically significant positive coefficient (11.227, p < 0.01) of climate risk (Climate risk) on resource misallocation (Abrupt), confirming that climate risk amplifies distortions in corporate internal capital markets.

**Table 5:** Resource allocation mechanism.

Variable	(1)	(2)
	AbRpt	Resilience
AbRpt		−0.001***
		(0.000)
Climate risk	11.227***	−0.013
	(0.943)	(0.011)
age	0.771***	−0.005*
	(0.222)	(0.003)
roe	−1.019	0.002
	(0.751)	(0.009)
size	−0.307***	0.012***
	(0.112)	(0.001)
lev	−1.744**	−0.036***
	(0.717)	(0.008)
top1	0.854	0.017*
	(0.746)	(0.009)
MB	0.825	−0.000
	(0.653)	(0.008)
growth	0.664	−0.003
	(0.614)	(0.007)
_cons	−0.380	0.141***
	(2.217)	(0.026)
Observations	2,053	2,053
R-squared	0.306	0.970

t statistics in parentheses.

* p < 0.1, ** p < 0.05, *** p < 0.01.

When both climate risk (Climate risk) and resource misallocation (Abrupt) are incorporated into the model: Resource misallocation (Abrupt) exhibits a significant negative coefficient (−0.001, p < 0.01) on Resilience, translating to a 0.1% decline in organizational resilience per unit increase in misallocation. The coefficient of climate risk (Climate risk) decreases from −0.029 in Table 4 Column (1) to an insignificant −0.013 in Table 5 Column (3), establishing complete mediation by resource misallocation. The indirect effect through this pathway is calculated as:11.227 × (−0.001) = −0.0112

This constitutes (−0.0112) / (−0.029) ≈ 38.62% of the total effect, indicating that climate risk reduces organizational resilience by 0.0112 units per unit increase via resource misallocation. Crucially, climate risk-induced capital market distortions (Abrupt) compel firms to prioritize resource allocation to high-carbon sectors (e.g., coal power supply), thereby crowding out investments in low-carbon transitions and creating a self-reinforcing cycle of “high-carbon investment→resource misallocation →high-carbon reinvestment”.

Combined with the 46.55% indirect effect from Table 4’s financing constraint pathway, these dual mechanisms collectively account for 85.17% of climate risk’s total suppressive effect on resilience, empirically validating both financial resource constraints and internal allocation distortions as transmission channels. Table 5 provides robust support for Hypothesis H2: Climate risk-driven resource misallocation generates financial lock-in effects that erode organizational resilience. Integrated with Table 4’s findings, these dual mediation pathways elucidate how high-carbon firms become ensnared in an “investment inertia→resource constraints→resilience degradation” closed loop, providing microeconomic evidence for climate transition policy design..

As illustrated in Table 6, under the external financing constraint path, the direct impact coefficient of climate risk on financing constraints is 0.0551, and the negative impact coefficient of financing constraints on corporate resilience is −0.2456. This finding aligns with the conclusions drawn in Table 4. The indirect effect was found to be −0.0135 at the 1% significance level, contributing 47.20% to the total effect. In the internal resource misallocation path, the coefficient for climate risk exacerbating resource misallocation is 11.2273, and the coefficient for resource misallocation inhibiting resilience is −0.0014 (same conclusion as Table 5). The indirect effect was found to be statistically significant at the 1% level at −0.0152, accounting for 53.12% of the total effect. The combination of these two paths results in complete mediation, and the higher contribution of the resource misallocation path underscores the notion that capital allocation distortion serves as the primary transmission mechanism.

**Table 6:** Sobel test results.

Statistic	External Financing Constraints (SA)	Internal Resource Misallocation (AbRpt)
Path Coefficients		
a coefficient (X → M)	0.0551***(0.0103)	11.2273***(0.9432)
b coefficient (M → Y)	−0.2456***(0.0231)	−0.0014** * (0.0003)
Effect Decomposition		
Indirect effect (a × b)	−0.0135***(0.0028)	−0.0152** * (0.0032)
Direct effect (X → Y)	−0.0151 (0.0107)	−0.0134 (0.0113)
Total effect (X → Y)	−0.0287***(0.0110)	−0.0287***(0.0110)
Test Statistics		
Sobel Z-value	−4.77***	−4.81***
Goodman-1 Z-value	−4.75***	−4.79***
Goodman-2 Z-value	−4.79***	−4.82***
Effect Interpretation		
Mediated effect proportion (%)	47.20%	53.12%
Indirect/Direct effect ratio	0.894	1.133

The Sobel test further substantiated the reliability of the fundamental regression through triple verification. Firstly, the Z-values of the external financing constraint path (−4.77, p < 0.01) and the internal resource misallocation path (−4.81, p < 0.01) both exceeded the 1% significance threshold. Secondly, the Z-values of the Goodman-1 and Goodman-2 tests were both significant (−4.The 75 to −4.82 range (p < 0.01) indicates the reliability of the indirect effect estimates. Furthermore, the direct effects of both paths are non-significant, which validates the mechanism through which climate risk is transmitted entirely via mediating variables. Contribution analysis provides a quantitative assessment of the significance of the paths. The resource misallocation path accounts for 53.12%, which is significantly higher than the financing constraint path at 47.20%. This finding suggests that the decline in capital allocation efficiency is the more critical transmission channel. This outcome serves to substantiate the statistical significance of the regression coefficients enumerated in Tables 4 and 5. Moreover, it unveils the target of policy intervention through effect decomposition. The necessity of prioritizing the optimization of internal resource allocation efficiency is revealed as a means to break the high-carbon lock-in.

The study employs a chain mediation effect test to examine the chain effect of external financing constraints on internal resource misallocation. It systematically investigates the transmission path of climate risk through external financing constraints (SA) → internal resource misallocation (AbRpt) → organizational resilience. The empirical evidence suggests a negative correlation between resource misallocation and organizational resilience (b = −0.001, p < 0.001). The chained indirect effect value, derived from 5,000 Bootstrap samples, is 0.0005 (95% CI [−0.003, 0.004]), indicating statistical insignificance (p = 0.778). This finding suggests that the transmission mechanism of financing constraints on resource misallocation has not yet established an effective pathway. One potential explanation for this phenomenon is that financing constraints may prompt corporate restructuring of resource architecture rather than passive misallocation.

### 4.3 The regulatory role of internal capital market

The validated mediation effect of Hypothesis 2 in Table 5 confirms that climate risk reduces corporate resilience through exacerbated efficiency losses (Abrupt). This mechanism likely originates from the long-term horizon of new energy transition investments: sampled firms exhibit an average 7.5-year investment cycle for low-carbon equipment, exacerbating short-term resource allocation distortions. Existing studies (such as Stein, 1997; Williamson, 1975) reveals the dual nature of internal capital markets in “efficiency-flexibility trade-offs”: although declining aggregate allocation efficiency may erode long-term competitiveness, the coordination advantage of internal markets can mitigate immediate shocks through rapid capital reallocation. To dissect this dual-effect mechanism, we focus on two interdependent regulatory pathways—business resource allocation and financial resource allocation. Specifically, climate risk triggers preferential capital distribution to existing high-carbon operations (business resource channel), while simultaneously distorting internal capital markets’ risk-bearing capacity for transitional investments (financial resource channel), thereby creating hysteresis effects in green financing. The subsequent analysis systematically examines how these dual channels interactively regulate corporate resilience under climate shocks.

#### 4.3.1 Internal capital market operations: Business resource allocation.

The regression results in column (2) of Table 7 reveal a coefficient of 0.031 for the interaction term between climate risk and post-decentralization Rptr, indicating that a one-unit increase in internal capital market activity reduces the negative impact of climate risk on corporate resilience by 0.031. This empirically validates the moderating mechanism whereby internal capital markets alleviate financing constraints by functioning as “short-term liquidity pools.” When external green credit is constrained, firms can rapidly reallocate resources through internal capital pools, with the standalone Rptr coefficient of 0.002 further indicating that proactive capital allocation not only emits positive market signals but also demonstrates risk-mitigation capabilities through internal resource mobilization, thereby buffering stock price volatility. The short-term relief mechanism essentially leverages administrative coordination advantages inherent to internal capital markets, utilizing fund transfers and asset restructuring to create strategic adjustment windows. These findings align with the regulatory mechanisms of internal capital market activity documented in Table 7. Importantly, while cross-period allocation through capital pools mitigates immediate shocks, it may exacerbate long-term efficiency losses, forming a theoretical nexus with the “efficiency-resilience trade-off” posited in Hypothesis 2.

**Table 7:** Regulating mechanism of internal capital market activity.

variable	(1)	(2)
	Resilience	Resilience
Climate risk	−0.029***	−0.024**
	(0.011)	(0.012)
Rptr		0.002***
		(0.001)
Climate risk**×**Rptr		0.031***
		(0.006)
age	−0.006**	−0.004
	(0.003)	(0.003)
roe	0.004	0.002
	(0.009)	(0.008)
size	0.012***	0.010***
	(0.001)	(0.001)
lev	−0.033***	−0.036***
	(0.008)	(0.008)
top1	0.015*	0.015*
	(0.009)	(0.008)
MB	−0.002	0.007
	(0.008)	(0.007)
growth	−0.004	0.001
	(0.007)	(0.007)
Constant	0.142***	0.154***
	(0.026)	(0.025)
Observations	2,053	2,053
R-squared	0.969	0.971
Year FE	Yes	Yes
Ins FE	Yes	Yes

Standard errors in parentheses.

*** p < 0.01, ** p < 0.05, * p < 0.1.

#### 4.3.2 Internal capital market operations: Financialized resource allocation.

The regression results in Table 8 show a reduced absolute value of the Climate risk coefficient alongside a statistically significant interaction term, demonstrating that financialization primarily amplifies climate risk impacts through moderating mechanisms. Specifically, the peer effect of financialization (Ind_Fin) in Model (2) reveals a centered interaction term (Climate risk × Ind_Fin) coefficient of −0.593, significant at the 1% level, indicating that each unit increase in climate risk depresses corporate resilience by an additional 0.593 units when financialization is present. This amplification mechanism operates through industry-wide herding effects: financial asset allocation within internal capital markets triggers collective imitation across sectors, fostering short-term profit-seeking behavior that magnifies climate risk vulnerabilities. Mechanically, this phenomenon arises as firms replicate peer-driven financial strategies to exploit internal capital pools, prioritizing liquidity management over long-term resilience-building. Crucially, while Table 8 confirms the moderating role of financialization peer effects, the induced herd behavior creates systemic fragility by synchronizing risk exposures across industries, thereby transforming firm-specific climate risks into sector-wide destabilization channels—a finding that extends Hypothesis 2’s efficiency-resilience trade-off framework to incorporate peer-driven amplification dynamics.

**Table 8:** The moderating mechanism of financialization peer effect.

Variable	(1)	(2)
	Resilience	Resilience
Climate risk	−0.029***	−0.023*
	(0.011)	(0.013)
Ind_Fin		−0.042**
		(0.020)
Climate risk×Ind_Fin		−0.593***
		(0.152)
age	−0.006**	−0.006**
	(0.003)	(0.003)
roe	0.004	0.004
	(0.009)	(0.009)
size	0.012***	0.011***
	(0.001)	(0.001)
lev	−0.033***	−0.031***
	(0.008)	(0.008)
top1	0.015*	0.013
	(0.009)	(0.009)
MB	−0.002	0.000
	(0.008)	(0.008)
growth	−0.004	−0.003
	(0.007)	(0.007)
Constant	0.142***	0.166***
	(0.026)	(0.026)
Observations	2,053	2,053
R-squared	0.969	0.970
Year FE	Yes	Yes
Ins FE	Yes	Yes

Standard errors in parentheses.

*** p < 0.01, ** p < 0.05, * p < 0.1.

## 5. Robustness test

### 5.1 Alternative measurement of climate risk variables

Given potential measurement biases in the text-based construction of the climate risk (Climate Risk) variable, we conduct robustness checks by introducing an alternative metric, Climate Risk2, which is derived from annual report text frequency statistics and calculated using the same methodological approach as the original variable. The results confirm the robustness of our findings: the core explanatory variable climateRisk2 exhibits a significantly negative coefficient (−0.036) in the benchmark model, while its one-period lagged term (L.Climate Risk2) maintains a persistent adverse effect (−0.031), demonstrating the temporal persistence of climate risk’s impact on corporate resilience. All models show strong explanatory power with R² values exceeding 0.96 and include controls for year and industry fixed effects, further validating the reliability of the main effect conclusion. This recalibration not only addresses potential measurement errors but also underscores the durability of climate risk’s influence, reinforcing the robustness of the study’s key insights.

### 5.2 Hausman test for model specification

To ensure the robustness of model selection, we conducted the Hausman test, which systematically evaluates the appropriateness of fixed versus random effects models. The test results strongly reject the random effects hypothesis (χ² = 437.50, p = 0.0000), indicating a systematic relationship between unobservable firm-level heterogeneity (individual effects) and explanatory variables, including climate risk. This finding confirms the superiority of the fixed effects model over the random effects model, as it effectively accounts for firm-specific unobservable characteristics that could bias the estimates. By controlling for these intrinsic firm-level factors, the fixed effects model provides more reliable and consistent estimates of the impact of climate risk on corporate resilience, further validating the robustness of the main effect results.

### 5.3 Endogeneity test: PSM

To address potential sample selection bias and endogeneity issues between climate risk mitigation (climate Risk) and organizational resilience (Resilience), we employ propensity score matching (PSM) as a robustness test. First, following Du Jian et al. (2023) [35], we construct a control group by generating a binary climate risk variable (set to 1 if a company’s climate risk exceeds the median, otherwise 0), thereby dividing firms into “high climate risk” and “low climate risk” groups. Using the previously defined control variables, we perform one-to-one nearest neighbor matching to ensure comparable distributions of matching variables between groups, simulating quasi-natural experimental conditions. Second, we estimate propensity scores using a logit model and validate the matching quality through balance tests, confirming no significant differences in covariates post-matching. Third, the regression results post-matching, as shown in Table 9, reveal a climate risk coefficient of −0.018, consistent in direction with the main model but with enhanced statistical significance. This confirms that the core findings are robust to sample selection issues, further supporting the reliability of the study’s conclusions.

**Table 9:** Endogeneity test based on propensity score matching.

Variable	(1)
	Resilience
Climate risk	−0.018***
	(0.004)
age	−0.010***
	(0.003)
roe	−0.017
	(0.011)
size	0.013***
	(0.002)
lev	−0.043***
	(0.011)
top1	0.012
	(0.011)
MB	0.001
	(0.010)
growth	−0.003
	(0.009)
Constant	0.146***
	(0.034)
Observations	1,136
R-squared	0.973
control	Yes
year FE	Yes
Ins FE	Yes

Standard errors in parentheses.

*** p < 0.01, ** p < 0.05, * p < 0.1.

## 6. Further analysis

### 6.1 Heterogeneous effects of climate risk types on resilience

To explore the differential impacts of climate risks, we categorize them into acute risks, chronic risks, and transition risks, with the first two falling under physical risks. We construct three sub-term sets for severe, chronic, and transition risks, estimating their respective indicators and regressing them against organizational resilience using models (4)-(6). The regression equations are specified as follows:


$${Resilience}_{i,t}=\alpha_{\text{0}}+\alpha_{\text{1}}{AcuteRisk}_{i,t}+\alpha_{\text{2}}{Controls}_{i,t}+\mu_{i}+\delta_{t}+\varepsilon_{i,t}$$ (4)

$${Resilience}_{i,t}=\alpha_{\text{0}}+\alpha_{\text{1}}{ChronicRisk}_{i,t}+\alpha_{\text{2}}{Controls}_{i,t}+\mu_{i}+\delta_{t}+\varepsilon_{i,t}$$ (5)

$${Resilience}_{i,t}=\alpha_{\text{0}}+\alpha_{\text{1}}{TransitRisk}_{i,t}+\alpha_{\text{2}}{Controls}_{i,t}+\mu_{i}+\delta_{t}+\varepsilon_{i,t}$$ (6)

The regression results in Table 10 reveal heterogeneous effects: Column (1) shows that acute risk (Acute Risk) has no significant impact on resilience, indirectly confirming that internal capital markets can buffer short-term climate shocks. Column (2) reports a significant negative coefficient for chronic risk (Chronic Risk) at −0.799 (p < 0.05), indicating that prolonged climate pressures substantially erode resilience. Column (3) demonstrates a weakly significant negative coefficient for transition risk (Transit Risk) at −0.034 (p < 0.1), suggesting that while carbon pricing and similar policies may initially suppress performance through demand shocks, they also create option value for technological adoption, resulting in a net hedging effect. These findings highlight the need for firms to leverage internal capital markets for risk mitigation, establish policy signal monitoring systems, and strategically manage transition risks to enhance long-term resilience.

**Table 10:** Heterogeneous effects of climate risk types on resilience.

Variable	(1)	(2)	(3)
	Resilience	Resilience	Resilience
Acute Risk	0.370		
	(0.604)		
Chronic Risk		−0.799**	
		(0.334)	
Transit Risk			−0.034*
			(0.018)
age	−0.005*	−0.006**	−0.005**
	(0.003)	(0.003)	(0.003)
roe	0.003	0.004	0.004
	(0.009)	(0.009)	(0.009)
size	0.011***	0.011***	0.012***
	(0.001)	(0.001)	(0.001)
lev	−0.033***	−0.032***	−0.033***
	(0.008)	(0.008)	(0.008)
top1	0.015*	0.013	0.015*
	(0.009)	(0.009)	(0.009)
MB	−0.002	−0.002	−0.001
	(0.008)	(0.008)	(0.008)
growth	−0.005	−0.005	−0.005
	(0.007)	(0.007)	(0.007)
Constant	0.149***	0.154***	0.138***
	(0.026)	(0.026)	(0.026)
Observations	2,053	2,053	2,053
R-squared	0.969	0.969	0.969
Year FE	Yes	Yes	Yes
Ins FE	Yes	Yes	Yes

Standard errors in parentheses.

*** p < 0.01, ** p < 0.05, * p < 0.1.

### 6.2 Climate risk and its impact on short- and long-term resilience

Drawing on the dual-cycle model of corporate resilience proposed by Flammer et al. (2021) [41], we disentangle the effects of external shocks into time decay effects (short-term) and path dependence effects (long-term). To measure short-term financial fluctuations, we use stock return volatility (SD, annual standard deviation), which captures the market’s immediate pricing response to sudden risks and the effectiveness of liquidity management and emergency mechanisms. For long-term performance, we employ the cumulative five-year sales revenue growth rate (PV). The regression results in Table 11 reveal distinct temporal impacts: Column (1) shows a weak and statistically insignificant coefficient for climate risk (0.011) on short-term financial volatility, suggesting that firms can buffer immediate climate risk effects through short-term adjustments such as cash flow management and risk hedging. In contrast, Column (2) demonstrates a significant negative coefficient for climate risk (−0.250, p < 0.01) on long-term performance growth, indicating that each unit increase in climate risk reduces cumulative sales revenue growth by 0.25 units. This underscores the cumulative threat posed by climate risks, including policy shifts and supply chain disruptions, to long-term corporate development. These findings highlight the need for differentiated resilience strategies: in the short term, firms should optimize internal resource allocation to stabilize profits and enhance risk resistance; in the long term, climate risk management must be integrated into strategic planning, supply chain resilience must be strengthened, and governance structures must be improved to mitigate cumulative impacts and unlock growth potential.

**Table 11:** Climate risk and its impact on short- and long-term resilience.

variable	(1)	(2)
	SD	PV
Climate risk	0.011	−0.250***
	(0.010)	(0.012)
age	0.007***	−0.001
	(0.002)	(0.003)
roe	−0.014*	0.304***
	(0.008)	(0.010)
size	−0.003***	0.001
	(0.001)	(0.001)
lev	0.052***	0.015
	(0.008)	(0.009)
top1	0.005	−0.022**
	(0.008)	(0.010)
MB	−0.075***	−0.006
	(0.007)	(0.009)
growth	0.014**	0.006
	(0.007)	(0.008)
Constant	0.196***	0.068**
	(0.024)	(0.029)
Observations	2,053	2,053
R-squared	0.398	0.515
Year FE	Yes	Yes
Ins FE	Yes	Yes

Standard errors in parentheses.

*** p < 0.01, ** p < 0.05, * p < 0.1.


$${SD}_{i,t}=\alpha_{\text{0}}+\alpha_{\text{1}}{Climate\;risk}_{i,t}+\alpha_{\text{2}}{Controls}_{i,t}+\mu_{i}+\delta_{t}+\varepsilon_{i,t}$$ (7)

$${PV}_{i,t}=\alpha_{\text{0}}+\alpha_{\text{1}}{Climate\;risk}_{i,t}+\alpha_{\text{2}}{Controls}_{i,t}+\mu_{i}+\delta_{t}+\varepsilon_{i,t}$$ (8)

## 7. Conclusions

This research investigates the dynamic impact of climate risk on organizational resilience in Chinese A-share listed high-carbon enterprises from 2008 to 2023, focusing on the transmission mechanisms of financial lock-in effects and the regulatory role of internal capital markets. The findings reveal that climate risk exacerbates financing constraints and internal capital misallocation, creating a “financial lock-in” effect that significantly undermines corporate resilience. Further analysis demonstrates asymmetric impacts across risk types: chronic physical risks exert more intense long-term erosion than transition risks and acute risks. While internal capital markets serve as a double-edged sword—mitigating short-term shocks through active resource allocation—over-reliance on financialized asset allocation amplifies long-term transition risks, compounded by industry-wide financialization’s herd contagion effects.

Based on these findings, the study proposes three policy recommendations:

Firstly, the establishment of a climate-resilient financial system is imperative to alleviate financing constraints experienced by enterprises. The development of transition finance tools is imperative for addressing the challenges posed by climate change. One potential solution is to allow enterprises to utilize carbon credits as eligible collateral for obtaining low-cost funding. Additionally, the establishment of a climate insurance securitization mechanism is crucial. This mechanism should encompass asset damage caused by physical risks, thereby expanding the coverage scope of catastrophe bonds and reducing the strain on insurance costs on cash flow. In the short term, the internal capital market can be utilized to provide short-term relief. A combination of “bridge loans plus green debt-to-equity swaps” can be designed to achieve this. This combination allows businesses to apply for policy-based bridge funding during the post-disaster recovery period. However, businesses must commit to allocating a proportion of the saved cash flow to a low-carbon technology account. This commitment is necessary to prevent resource misallocation and the entrenchment of high-carbon pathways.
Secondly, optimize corporate capital allocation frameworks by enhancing internal governance and supervision. Enterprises are advised to enhance their internal governance, strengthen their internal capital supervision, and optimize their resource allocation. It is recommended that listed companies disclose the proportion of climate risk hedging positions and low-carbon technology investments. Furthermore, enterprises with financial asset allocations exceeding a certain proportion of net assets should be subject to ESG rating downgrades. Enterprises should establish independently accounted low-carbon innovation funds to ensure that internal cash flows are used for cutting-edge technology research and development. Concurrently, the utilization of blockchain technology for the comprehensive oversight of green credit flows should be investigated.
Thirdly, create an industry-wide collaborative governance platform led by the Ministry of Ecology and Environment. It is recommended that the Ministry of Ecology and Environment and the China Securities Regulatory Commission collaborate in the construction of an “industry resilience rating system.” The implementation of this system would entail the establishment of a tiered carbon quota and subsidy reduction system for enterprises. The objective of this system is to compel the entire industry chain to enhance its resilience. It is imperative to establish a “transformation progress red and black list” for high-carbon industries. Furthermore, new credit should be suspended for enterprises with a financialization level that significantly exceeds the industry average. Additionally, initiative capital misallocation must be curbed.

The study acknowledges limitations, including potential underestimation of hidden risks due to reliance on text-based climate risk measures and data constraints preventing deeper analysis of internal market allocation dynamics and the interplay between green technology investment and financial lock-in. Future research could leverage natural experiments to further explore strategies for breaking financial lock-in and enhancing climate resilience.

## Supporting information

S1 Data It contains all 2053 observations of this study, including the variables involved in the paper. (XLSX)
